# The Track of Brain Activity during the Observation of TV Commercials with the High-Resolution EEG Technology

**DOI:** 10.1155/2009/652078

**Published:** 2009-06-22

**Authors:** Laura Astolfi, Giovanni Vecchiato, Fabrizio De Vico Fallani, Serenella Salinari, Febo Cincotti, Fabio Aloise, Donatella Mattia, Maria Grazia Marciani, Luigi Bianchi, Ramon Soranzo, Fabio Babiloni

**Affiliations:** ^1^IRCCS, Fondazione Santa Lucia, 00179 Rome, Italy; ^2^Dipartimento di Informatica e Sistemistica, Università di Roma “La Sapienza”, 00185 Rome, Italy; ^3^Department of Physiology and Pharmacology, University of Rome “La Sapienza”, 00185 Rome, Italy; ^4^Department of Neuroscience, University of Rome “Tor Vergata”, 00100 Rome, Italy

## Abstract

We estimate cortical activity in normal subjects during the observation of TV commercials inserted within a movie by using high-resolution EEG techniques. The brain activity was evaluated in both time and frequency domains by solving the associate inverse problem of EEG with the use of realistic head models. In particular, we recover statistically significant information about cortical areas engaged by particular scenes inserted within the TV commercial proposed with respect to the brain activity estimated while watching a documentary. Results obtained in the population investigated suggest that the statistically significant brain activity during the observation of the TV commercial was mainly concentrated in frontoparietal cortical areas, roughly coincident with the Brodmann areas 8, 9, and 7, in the analyzed population.

## 1. Introduction

In the recent years, researchers have begun to use neuroimaging tools to examine human behaviour in economic games and decision making between different commercial advertisements. This field is known as Neuromarketing. Principal issue of this branch is to understand mechanisms underlying customer's engagement with brand or company advertised [1–3]. In particular, the question is to explain how the exposure of a message, made up of images, text, and audio, is able to trigger in the consumer mind persisting stimuli leading to an interest, preference, purchase, and repurchase of a given product. In the same way they try to explain how video's emotional contents work after observing a humanitarian TV spot. Since marketers need to be reassured that a new advertising campaign will work before airing it, they trust an advertising test made on small groups of people which allows them to decide whether promoting the campaign or not. This test consists in an interview asking about the likeability, emotional involvement, persuasion, and intention to purchase.

In the last decades, several authors have investigated the capability of subjects to memorize and retrieve sensible “commercial” information observed during a TV spot [4–9]. The most used neuroimaging tool to track the brain response to the commercial advertisements is the functional Magnetic Resonance Imaging (fMRI) technique, able to return the profile of brain areas that elicited increased blood flow during the task when compared to a resting state. However, there are precise limitations in the actual state of the art of this technique. Essentially, the main limitation is linked to the insufficient temporal resolution of fMRI. In fact, temporal resolution of hundred of milliseconds or less is necessary to track the shifts of brain activity closely related to the processing of visual and acoustic stimuli provided by the fast moving of visual commercial spots. 

For this reason other authors also adopt different tools such as magnetoencephalography (MEG). This technique is sensitive to changes of magnetic fields that are induced by the electrical brain activity, and it is able to detect rapid changes of the neural activity on a temporal scale of milliseconds and on a spatial scale of centimetres. 

It is worth noticing that the past several studies also used electroencephalography (EEG) as brain imaging tool for the analysis of brain activity during the observation of TV commercials. However, at that time, EEG limitations in spatial resolution due to an insufficient number of electrodes used as well as to the limited processing capabilities were responsible for a series of inconclusive and fragmented observations of these phenomena. Nowadays, high-resolution EEG technology has been developed to enhance the poor spatial information content of the EEG activity in order to detect the brain activity with a spatial resolution of a squared centimetre and the unsurpassed time resolution of milliseconds [10–14]. 

The purpose of this paper is to illustrate the potential of the high-resolution EEG techniques when applied to the analysis of brain activity related to the observation of TV commercials. In particular, we would like to describe how by using appropriate statistical analysis it could be possible to recover significant information about cortical areas engaged by particular scenes inserted within the TV commercial analyzed. 

In order to do that, we recorded a series of normal subjects with high resolution EEG techniques during the observation of a documentary in which an interruption was generated. The subjects were not aware of the aim of the study. The brain activity was evaluated in both time and frequency domains by solving the associate inverse problem of EEG with the use of realistic head models. 

Cortical activity estimated during the observation of the TV commercial was then compared with the brain activity computed in the analyzed population during the observation of the documentary.

## 2. Materials and Methods

### 2.1. Experimental Design

The whole dataset is composed by EEG registrations of 13 healthy subjects (mean age 30 ± 4 years) watching a documentary of 30 minutes intermingled by a TV commercial (see [15]). Each subject is exposed to the observation of a same documentary. The subjects were not aware of the aim of the recording, and they only knew to pay attention to the material showed on the screen during the entire 30 minutes. The TV commercial, whose length was 30 seconds, was inserted at the middle of the documentary. Such commercial was realized for a popular brand of beer in Italy, that was on-air on the national TV channels on the days in which the experiment was realized. After the EEG registration each subject was recalled in laboratory, where an interview was performed. In such interview, the subjects were asked if they usually drink beer or light alcohol at least once per week. If yes, subjects were considered within the dataset of “drinkers” in opposition to the dataset of “no drinkers.” In order to increase the sensitivity of the analysis performed, only the EEG spectral analysis for the “drinkers” was analyzed and presented here. 

The hypothesis was that the TV commercial could be better followed by a class of subjects who usually drink beer instead that from other “nondrinkers” subjects.

### 2.2. High-Resolution EEG: Recordings and Processing Techniques

High-resolution EEG technologies have been developed to enhance the poor spatial information content of the EEG activity [10, 16, 17]. Basically, these techniques involve the use of a large number (64–256) of scalp electrodes. In addition, high-resolution EEG techniques rely on realistic MRI-constructed head models [18, 19] and spatial deconvolution estimations, which are usually computed by solving a linear-inverse problem based on Boundary-Element Mathematics [13, 20]. Subjects were comfortably seated on a reclining chair, in an electrically shielded, dimly lit room. A 64-channel EEG system (BrainAmp, Brainproducts GmbH, Germany) was used to record electrical potentials by means of an electrode cap, accordingly to an extension of the 10–20 international system. In the present paper, the cortical activity was estimated from scalp EEG recordings by using realistic head models whose cortical surface consisted of about 5000 triangles uniformly disposed. The current density estimation of each one of the triangle, which represents the electrical dipole of the underlying neuronal population, was computed by solving the linear-inverse problem according to the techniques described in the previous papers [14, 15, 21]. 

Thus, a time-varying waveform relative to the estimated current density activity at each single triangle of the modeled cortical surface was obtained. Such waveform was then subjected to the time-varying spectral analysis by computing the spectral power in the different frequency bands usually employed in EEG analysis, that is, theta (4–7 Hz), alpha (8–12 Hz), beta (13–24 Hz), and gamma (24–45 Hz). 

Although we estimated brain activity in all the described frequency bands, in the following we presented those related to theta and alpha frequency bands. In fact, in the EEG literature, these frequency bands have been suggested to be maximally responsive during the observation and the memorization tasks when compared to the beta and gamma bands [22]. 

In each subject recorded, the statistical significance of the spectral values during the observation of the TV commercials was then measured against the activity evaluated during the observation of the documentary for the same subject. This was obtained by computing a time-varying z-score variable for each subject and for each dipole placed on the cortical mantle in the analyzed frequency band. The mean and the standard deviation for such z-score variable was estimated in the documentary period, while the time-varying values of the spectral power in the theta band during the observation of the TV commercial for each dipole were employed. 

In order to present these results relative to the experimental conditions for the entire population, we needed a common cortical representation to map the different activated areas of each subject. For this purpose we used the average brain model available from the McGill University website. In this way we were able to display the cortical areas that are statistically significant activated during different experimental conditions in all subjects analyzed. In fact, we highlighted in yellow a voxel of the average brain model if it was a cortical site in which a statistical significant variation of the spectral power between the experimental conditions was found in all the subjects; if such brain voxel was statistically significant in all but one of the subjects analyzed, we depicted it in red. In all the other cases the voxel was represented with a gray colour. 

By construction, the analyzed maps are then relative to the evolution of the time-cortical activity of the spectral power in the theta band. However, only the statistical significant variation of such spectral power when compared to the documentary period was highlighted in colour. The use of z-score will allow us to have a variable that can be averaged and can be used to synthesize the results of the entire population investigated.

## 3. Results

Of the 13 subjects recorded, only seven are “drinkers.” Hence, the successive analysis and results are presented for seven of such subjects.

We summarized all results for the “drinkers” group in a series of figures showing the statistically significant differences of cortical activation concerning this dataset in the theta frequency band (4–7 Hz). Data regarding the alpha frequency band (8–12 Hz) were equivalent to the theta band and for this reason not shown here. Our figures are formed by a series of subsequent panels each containing two images: the upper one represents a frame of the TV commercial while the lower one displays the corresponding mean brain activity. In particular, the image at the bottom of the panel shows four different views of the average brain model organized in two rows: the upper row comprises the front and left perspective while the lower one the rear and right brain perspective. The temporal axes beat the time of the commercial.

**Figure 1:**
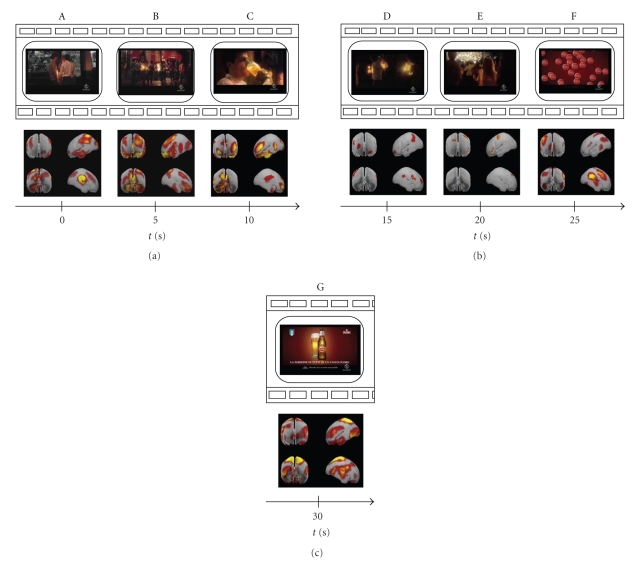
Tracking of the mean cortical activity of the group of “drinkers” in the theta frequency band spot. The statistical significant activity in this population is shown in seven panels (A–G), each representing subsequent film segments of a TV spot with corresponding brain activity. Temporal axes beat the spot time every 5 seconds: in this way panel A represents the first frame of the commercial while panel G shows the last one. This example illustrates how it is possible to track human cortical activity by means of the highresolution EEG technique.

**Figure 2:**
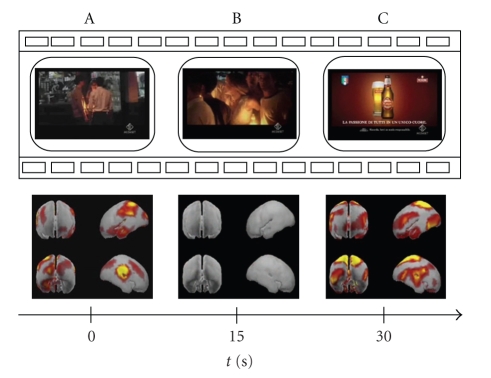
Tracking of the mean cortical activity of the group of “drinkers” in the theta frequency band spot. The statistical significant activity in this population is shown in 3 panels (A–C), each representing subsequent film segments of a TV spot with corresponding brain activity. Temporal axes beat the spot in correspondence of the beginning (A), the middle (B), and the end (C) of the entire film sequence.

In Figure 1  we present a first series of 7 film segments spanning the whole length of a certain TV spot. Frames are taken each 5 seconds from the beginning of the clip. In such a way panel A represents the first frame of the commercial while panel G shows the last one. By examining this strip it results evident how the temporal evolution of the mean cortical activity changes according to the images viewed by the subjects. In particular, an enhancement of cerebral activity is suggested by the result of the application of the statistic tests at the beginning and at the end of the videoclip presented. In fact, from the lower row of the figures, it is possible to observe how in the middle film segments very restricted areas provide statistically significant differences when compared to the ones watched at the beginning and at the end of the commercial. This drastic change of activity is more evident in Figure 2. The present figure is composed by 3 panels representing the first (panel A), the middle (B), and the last (C) frame of the TV spot, respectively. The corresponding mean cortical activity completes each panel of the figure. By observing these three images it is clear how the middle part of the commercial is characterized by cerebral zones displaying no statistical differences across ROIs, while there are two peaks of activity at the beginning and at the end of the clip.

**Figure 3:**
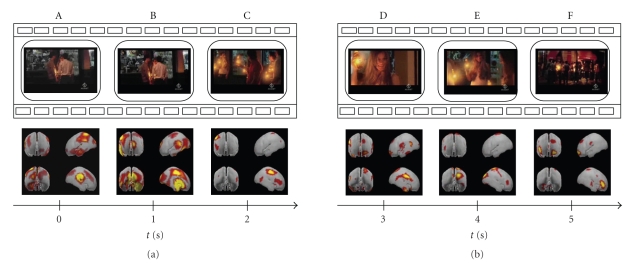
Tracking of the mean cortical activity in the theta frequency band of the first 5 seconds of the commercial spot. The statistical significant activity in this population is shown in six panels (A–F), each representing subsequent film segments of a TV spot with corresponding brain activity. Temporal axes beat the spot time every second: in this way panel A represents the first frame of the commercial while panel F shows the film segment shown after 5 seconds from the beginning.

**Figure 4:**
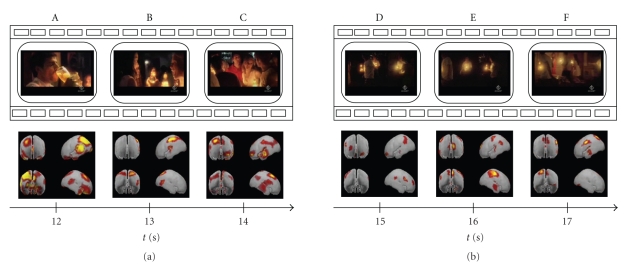
Tracking of the mean cortical activity of seven drinkers in the theta frequency band of the central 5 seconds of the commercial spot. The statistical significant activity in this population is shown in six panels (A–F), each representing subsequent film segments of a TV spot with corresponding brain activity. Temporal axes beat the spot time every second: in this way panel A represents the film segment after 12 seconds from the beginning of the commercial; panel F shows the film segment after 17 seconds.

**Figure 5:**
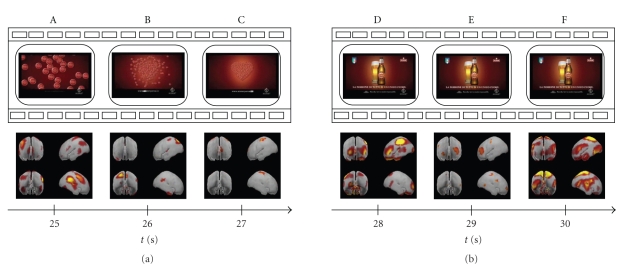
Tracking of the mean cortical activity of the last 5 seconds of the commercial spot. The statistical significant activity in this population is shown in six panels (A–F), each representing subsequent film segments of a TV spot with corresponding brain activity. Temporal axes beat the spot time every second: in this way panel F represents the last film segment of the commercial; panel A shows the film segment after 25 seconds from the beginning.

The analysis of the temporal evolution of the brain activity has been performed even on shorter intervals in order to track its variations in closer time instants. Subsequent Figures 3, 4, and 5  follow the cerebral activity with a higher temporal resolution. Time intervals spanned in the following figures correspond to the first 5 seconds (Figure 3), middle 5 seconds (Figure 4), and last 5 seconds of the commercial (Figure 5), respectively. These examples show how it is possible to catch statistically significant differences in the activation of cortical areas even reducing the time interval of interest.

## 4. Discussion

Thanks to the high resolution EEG techniques we tracked subjects' brain activity during visualization of the commercials: in such manner it is possible to obtain a global measure of the reconstructed cortical signals by means of a simple graphic tool which allows us to distinguish the activity of different cortical areas. The above-mentioned results allow us to comment temporal and spatial events observed. 

In fact, the observed phenomena suggest an active role of the prefrontal and parietal areas in the coding of the information possibly retained by the users from the TV commercials. A statistical increase of EEG spectral power in the prefrontal (namely, BA 8, 9) and parietal areas is in agreement with the suggested role of these regions during the transfer of sensory percepts from short-term memory to long-term memory storage. The results suggest a strong prevalence of a ‘common’ prefrontal bilateral (involving BA 8 and 9) activity in all the subjects analyzed during the observation of the TV commercials. In addition a stronger engagement of the left frontal areas has been noted, in agreement with the HERA model [23] in which such hemisphere plays a decisive role during the encoding phase of information from the short-term memory to the long-term memory, whereas the right hemisphere plays a role in the retrieval of such information. It must be noted, however, that the role of the right cortices in storing images has been also recognized for many years in neuroscience [2, 24].

As presented in the previous works performed both with EEG analysis and MEG recordings [5, 15], the observed phenomena suggest an active role of the prefrontal and parietal areas in coding of the information that will be retained by users from the TV commercials. In particular, activations of these cortical areas can be associated with attentional and memorization processes. As shown in the previous figures, peaks of activity emerge at the beginning and at the end of clip (Figures 1, 2). In these periods subjects' attention is more focused on what he/she sees, in particular when they watch scenes showing meeting moments (such as panels A, B in Figure 3) and the advertised product (panels D and F of Figure 5). Instead, in the middle of the TV clip, we observed a peak of activity only when subjects watch a person utilizing the advertised product (such as a beer in panel A of Figure 4). These processes could reflect memorization of significant frames' sequence which would help the subject to understand the whole video clip and messages provided. Climax of this elaboration will be achieved in the last film segments of the sequence when the meaning of the commercial will be completely understood (last panel of Figure 5).

The present paper intends to stress the useful properties of the high-resolution EEG technologies. In particular this tool is able to help us in observing and analysing the temporal trend of the cortical activities thanks to a high temporal and spatial resolution. These features allow us to distinguish a certain precision changes of activation of ROIs corresponding to different cortical areas, by means of a graphical representation on an average brain model. Our analysis focused the attention on tracking human brain activity with different time resolution, all offering the same spatial resolution able to discriminate activation's intensity of Brodmann areas.

The reconstruction of the cortical activity by means of the high resolution EEG technique [25–30] and by combining the above statistic treatment of our data allowed us to track subjects' brain activity during visualization of the commercials. In such a way for each film segment of a clip it was possible to distinguish cortical areas that were significantly activated when compared to the observation of the documentary. This could be useful in the evaluation of the cortical responses to particularly type of visual solicitations, performed by film or commercial clips, that at the moment is a largely unexplored field by neuroscience.
